# Circulating zinc levels and cardiometabolic risk-related variables in adults

**DOI:** 10.7705/biomedica.6882

**Published:** 2024-05-31

**Authors:** Milton Fabián Suárez-Ortegón, Alejandra Arbeláez, José Guillermo Ortega-Ávila, Mildrey Mosquera

**Affiliations:** 1 Departamento de Alimentación y Nutrición, Facultad de Ciencias de La Salud, Pontificia Universidad Javeriana, Seccional Cali, Cali, Colombia Pontificia Universidad Javeriana Departamento de Alimentación y Nutrición Facultad de Ciencias de La Salud Pontificia Universidad Javeriana Cali Colombia; 2 Grupo de Nutrición, Universidad del Valle, Cali, Colombia Universidad del Valle Grupo de Nutrición Universidad del Valle Cali Colombia; 3 Departamento de Ciencias Básicas, Facultad de Ciencias de La Salud, Pontificia Universidad Javeriana, Seccional Cali, Cali, Colombia Pontificia Universidad Javeriana Departamento de Ciencias Básicas Facultad de Ciencias de La Salud Pontificia Universidad Javeriana Cali Colombia; 4 Departamento de Ciencias Fisiológicas, Universidad del Valle, Cali, Colombia Universidad del Valle Departamento de Ciencias Fisiológicas Universidad del Valle Cali Colombia

**Keywords:** Zinc, heart disease risk factors, triglycerides, micronutrients., zinc, factores de riesgo de enfermedad cardiaca, trigliceridos, micronutrientes.

## Abstract

**Introduction.:**

Altered serum zinc levels, lower and higher than values in healthy controls, have been observed in individuals affected by non-communicable chronic diseases. However, to date, studies describing potential determinants of zinc levels in general populations free of chronic diseases appear to be limited.

**Objective.:**

To evaluate whether nutrient intake, biochemical and clinical measures, lifestyle, and family history of cardio-metabolic diseases are independently associated with zinc levels in apparently healthy individuals.

**Materials and methods.:**

We evaluated 239 healthy subjects. Serum zinc was measured via flame atomic absorption spectrometry, and the remaining biochemical markers were assessed using enzymatic colorimetric methods. Standard techniques were employed to quantify waist circumference, height, and weight. Body fat was measured via bioimpedance, and blood pressure was measured using digital sphygmomanometers. We applied a survey to record the personal and family history of non-communicable chronic diseases, and nutrient intake was estimated using the 24-hour recall method.

**Results.:**

Women had lower serum zinc levels than men. In multivariate analyzes, total fat intake (β = -0.15; standard error = 0.03; p < 0.001), plasma log-triglycerides (β = -10.18; standard error = 3.9; p = 0.010), and female gender (β = -6.81; standard error = 3.3; p = 0.043) were significant predictors for serum zinc levels. Zinc intake was not significantly related to serum zinc in univariate and multivariate analyses.

**Conclusions.:**

Variables related to cardiometabolic risk, such as plasma triglyceride levels and total fat intake, were associated with serum zinc levels in individuals without a diagnosis of chronic or infectious/inflammatory diseases. Further studies are required to confirm our findings and to evaluate possible biological mechanisms for these relationships.

## Resumen

**Introducción.:**

Se han observado niveles séricos alterados de zinc, más altos o más bajos, en personas afectadas por enfermedades crónicas no transmisibles. Sin embargo, la información sobre determinantes de zinc sérico en poblaciones sin enfermedad crónica es muy limitada.

**Objetivo.:**

Evaluar si la ingestión de nutrientes, las medidas bioquímicas y clínicas, el estilo de vida y los antecedentes familiares de las enfermedades cardiometabólicas están asociados de forma independiente con los niveles de zinc en individuos aparentemente sanos.

**Materiales y métodos.:**

Se evaluaron 239 sujetos sanos. El zinc sérico se midió por espectrometría de absorción atómica de llama y el resto de los marcadores bioquímicos por métodos enzimáticos-colorimétricos. Se utilizaron técnicas estándar para medir la antropometría. Se aplicó una encuesta para registrar antecedentes personales y familiares, y se estimó el consumo de nutrientes por recordatorio de 24 horas.

**Resultados.:**

Las mujeres tenían niveles séricos de zinc más bajos que los hombres. En los análisis multivariados, la ingestión total de grasas (β = -0,15; error estándar = 0,03; p < 0,001), los triglicéridos plasmáticos (β = -10,18; error estándar = 3,9; p = 0,010), y el sexo femenino (β = -6,81; error estándar = 3.3; p = 0,043) fueron predictores significativos de los niveles séricos de zinc. La ingestión de zinc no estuvo significativamente relacionada con el zinc sérico en los análisis univariados y multivariados.

**Conclusiones.:**

Las variables relacionadas con el riesgo cardiometabólico como los niveles de triglicéridos y la ingestión total de grasas se asociaron con los niveles de zinc en individuos sin diagnóstico de enfermedades crónicas o infecciosas-inflamatorias. Se requieren más estudios para confirmar estos hallazgos, así como la evaluación de los posibles mecanismos biológicos de estas relaciones.

Zinc is the most abundant intracellular trace element, serving as a structural and functional component in many enzymes involved in physiological functions such as cell division, growth, immune response, gene expression, sexual reproduction, and antioxidant defense, among others ^1^. Although zinc status has been studied concerning nutritional deficiency, in recent years, interest in zinc and its relationship with cardiometabolic diseases, such as type 2 diabetes and cardiovascular conditions, has grown, but results are still conflicting.

A systematic review and meta-analysis reported a positive association between serum or blood zinc and type 2 diabetes, presumably due to disturbed body zinc distribution ^2^. In contrast, Hennigar *et al*. did not find a significant difference in serum zinc concentration between individuals with and without diabetes in populations from the National Health and Nutrition Examination Survey (NHANES) 2011-2014 ^3^. Meanwhile, *in vitro* evidence shows zinc deficiency may lead to alterations in insulin secretion and predispose to type 2 diabetes ^4^^,^^5^.

However, studies *in vivo* (in humans) on circulating blood zinc and type 2 diabetes are limited, with most employing cross-sectional designs ^2^. Similarly, accumulated observations in cross-sectional studies have revealed a trend toward lower zinc status in individuals affected by cardiovascular diseases compared to controls ^6^. Additional reverse causation between cardiometabolic diseases and zinc is highly plausible as metabolic alterations inherent to cardiometabolic diseases, such as inflammation and increased oxidative stress, could also impact zinc balance via up-regulation of metallothioneins, proteins that capture zinc ions and impede their distribution in the body ^1^.

To date, studies describing potential determinants of zinc levels in general populations free of chronic diseases appear to be limited. Thus, we conducted a study to assess whether metabolic, nutritional, clinical, and lifestyle variables, as well as a family history of cardio-metabolic diseases, could be independently associated with zinc levels in apparently healthy individuals.

## Materials and methods

### Subjects

This study utilized available data from a project focusing on iron status, insulin resistance, and type 2 diabetes. The project maintains a bank of participants’ blood serum preserved at -70°C. Serum zinc measurements were successfully conducted in 239 out of 245 samples based on a feasible volume for the laboratory technique. The original project employed a convenience sample, with participants voluntarily recruited from the staff of a hospital, a university, a governmental health department, and a supermarket chain in Cali, Colombia, in response to research advertisements. The final sample comprised apparently healthy subjects (120 men, 119 women) aged 25-64.

To ensure a healthy condition and avoid confounding factors in serum zinc, lipid profile variables, insulin, glucose, or high sensitivity C-reactive protein (hs-CRP) levels, we excluded individuals with clinically significant hepatic, neurological, or endocrinologic diseases; cardio-metabolic diseases (except class I obesity) or another systemic disease; tobacco use; long-term multivitamin or vitamin supplement consumption (two or more days per week in the last six months); therapy with drugs to lower lipid or glycemia levels; and evidence of acute or chronic inflammatory or infective diseases.

The *Universidad del Valle* Research Ethics Committee approved the study (Permission number: 0016-07), and all participants provided written informed consent.

### Clinical measurements

Trained researchers measured waist circumference, height, and weight using standard techniques by Lohman ^7^^,^^8^ and calculated body mass index (BMI). Additionally, body fat percentages were estimated using bioimpedance equipment (Omron®), and blood pressure was quantified using digital sphygmomanometers (Omron®).

### Biochemical markers

The following biochemical markers were determined: Glycemia, plasma triglycerides, total cholesterol, and high-density lipoprotein cholesterol (HDL-C) (direct method) by a colorimetric assay (Biosystems®, Spain); insulin levels by chemiluminescence test (kit IMMULITE 1000, San José, CA), and levels of hs-CRP by turbidimetry (Biosystems®, Spain). An autoanalyzer A-15 was used (Biosystems®, Spain) to performed previous assays. Low-density lipoprotein cholesterol (LDL-C) was calculated using the Friedewald equation [total cholesterol - HDL-C - (triglycerides / 5)] ^9^. The Homeostatic Model Assessment of insulin resistance (HOMA-IR) index was computed as glucose mmol/L x insulin mU/mL / 22.5) ^10^.

The flame atomic absorption spectrometry method, standardized by Smith *et al*., was employed to measure serum zinc ^11^. To analyze zinc levels, we cleaned the test tubes with 0.1% nitric acid to ensure that the samples were not contaminated. To analyze Zn levels, plasma samples were diluted fivefold, mixing 500 pl of plasma with 2 ml of deionized water. The spectrometer Shimadzu AA-7000 aspirated the zinc calibrators in sequence -starting from the least to the most concentrated- until it achieved stable readings. The obtained values were used to create calibration curves through least squares regression fitting, which helped to extrapolate sample concentrations. The coefficient of variation was 5.4%, and the recovery percentage was 106.6%.

### Dietary intake and personal and family history

Food intake was estimated via the 24-hour dietary recall method using the food composition table of the Center of Nutritional Service elaborated by Quintero and Escobar ^12^. All the nutrients available in the table were evaluated. The dietary recalls were conducted on one day, either Thursday or Friday, the scheduled days for the complete evaluation of the recruited individuals. Similarly, a survey to record personal data and cardio-metabolic disease history in siblings, aunts/uncles, parents, and grandparents was applied by trained interviewers. The physical activity was recorded based on whether individuals had vigorous or moderate physical activity (at least three sessions of 30 minutes per week). The absence of physical activity was defined as not having any sessions per week.

### Data analysis

The analyses were carried out in the whole group without any stratification. In the case of non-Gaussian distribution, studied variables were reported as median and interquartile range, while mean and standard deviation were used for normally distributed variables. The relationship between potential determinants and serum zinc levels was tested via linear regression. Multiple linear regression models were built to explain serum zinc levels. Initially, potential determinants were explored through a preliminary univariate analysis, where variables with a p value < 0.10 entered the multiple explanatory model ^13^. In these multiple models, a p value < 0.05 was considered significant. Variables not normally distributed were log- transformed before inclusion in the linear regression analyses (intakes of carbohydrates, ascorbic acid, vitamin A, zinc, and iron; calories per day; HOMA-IR index; hs-CRP; and plasma triglycerides).

Additionally, a linear regression analysis was conducted, wherein the built multivariable explanatory model of serum zinc levels was further adjusted for a set of covariates (potential confounders or variables influencing the predictors-outcome relationship), regardless of the statistical significance of these covariates as predictors of zinc levels. The planned covariates included age, sex, hs-CRP levels, total intake (calories per day), BMI, and the absence of physical activity per week. The inclusion of a covariate was omitted if it was already part of the multivariable explanatory model. All analyses were performed using Stata^TM^, version 10.0, software.

## Results

Table 1 describes the studied population. The participants were adults with a mean age of 45.5 years, and a BMI falling within the overweight range. Among the individuals, 110 (46%) were overweight, and 48 (20%) had obesity class I. Average LDL-C and plasma triglyceride levels were in the upper reference range, and a family history of hypertension was the most prevalent cardio-metabolic disease antecedent.

**Table 1:** Description of the studied population (N = 239)

Age (years)^1^		45.5 ± 7.7
Clinical variables		
	BMI (kg/m2) ^1^	26.1 ± 3.7
	WC (cm)1	80.5 ± 10.4
	Body fat (%)^1^	31.7 ± 10.0
	SBP (mm Hg)^1^	117 ± 16.4
	DBP (mm Hg)^1^	74 ± 11.3
	Menopausal women, n (%)	50 (20.9)
Biochemical variables		
	Zinc (pg/dl)^1^	76.6 ± 13.5
	Cholesterol (mg/dl)^1^	197 ± 34.1
	HDL-C (mg/dl)^1^	48 ± 11.1
	LDL-C (mg/dl)^1^	115 ± 29.1
	Log-l Plasma triglycerides (mg/dl)^2^	131 (94-196)
	Log-Glucose (mg/dl)	88 (83-93)
	Insulin (mU/ml)	8.4 (5.7-13.3)
	Log-hs-CRP (mg/L)^2^	1.5 (1.1-2.4)
	Log-HOMA-IR2	1.7 (1.1-2.8)
Dietary intake		
	Total energy intake (cal/day)	1,859.3 (1,555.5-2,111.8)^2^
	Total fat (g/day)^1^	89.5 ± 27.7
	Carbohydrates (g/day)	197.6 (163.0- 51.3) ^2^
	Protein (g/day)^1^	65.9 ± 25.7
	Calcium (mg/day)^2^	366.5 (204.6-543.7)
	Iron (mg/day)^2^	9.1 (6.8-12.4)
	Zinc (mg/day)^2^	6.9 (4.3-10.1)
	Vitamin A (mg/day)^2^	406.3 (256.6-712.6)
	Vitamin C (mg/day)^2^	58.6 (37.5-115.4)
	Copper (mg/day)^2^	0.5 (0.3-0.7)
Family history of cardiometabolic disease		
	Myocardial infarction, n (%)	87 (36.4)
	Stroke, n (%)	42 (17.6)
	Dyslipidemia, n (%)	95 (39.7)
	Diabetes, n (%)	111 (46.4)
	Hypertension, n (%)	140 (58.6)
	Obesity, n(%)	81 (33.9)
Lifestyle variables		
	No physical activity peer week, n (%)	118 (49.4)

WC: Waist circumference; BMI: Body mass index; hs- CRP: High sensitivity C reactive protein; HDL-C, High-density lipoprotein cholesterol; LDL-C: Low- density lipoprotein cholesterol; SBP: Systolic blood pressure; DBP: Diastolic blood pressure; HOMA-IR: Homeostatic Model Assessment of insulin resistance

^1^ Mean ± standard deviation

^2^ Median (interquartile range)

The univariate analysis showed significant negative associations (p < 0.1) with serum zinc levels, except for log-plasma triglyceride levels and glycemia, which showed positive relationships (table 2). Intakes of minerals, including zinc itself, were not found to be associated with serum zinc levels. Female sex was inversely associated with serum zinc levels. Log-hs-CRP levels, self-reported family history of obesity, and total fat intake were also related to serum levels (p < 0.1).

**Table 2:** Univariate analysis With serum zinc levels as dependent variable in the whole group and by gender

Variable		**β coefficient**	Standard error	p value
Clinical variables				
	BMI	-0.18036	0.239	0.452
	WC	0.13669	0.083	0.104
	% Body fat	-0.27333	0.087	0.002
	SBP	0.01147	0.053	0.831
	DBP	-0.01801	0.077	0.817
	Menopause	2.42550	2.422	0.318
Biochemical variables				
	Cholesterol	0.02452	0.025	0.340
	HDL-C	-0.09298	0.078	0.239
	LDL-C	-0.03552	0.030	0.243
	Log-Plasma triglycerides	14.3445	3.610	0.00 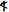
	Log-Glucose	41.2479	19.189	0.003
	Insulin	0.91705	3.381	0.786
	Log-HOMA	1.80126	3.176	0.571
	Log-hs-CRP	-0.50104	3.042	0.034
Dietary intake				
	Calories	-12.07119	7.711	0.116
	Total fat	-0.14774	0.030	0.00 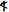
	Carbohydrates	5.92232	5.496	0.282
	Protein	0.02194	0.034	0.524
	Calcium	3.49406	2.660	0.190
	Iron	-1.03839	4.827	0.830
	Zinc	0.06269	0.190	0.742
	Log-Vitamin A	1.69669	1.900	0.393
	Log-Vitamin C	1.66690	2.422	0.492
	Copper	0.57591	1.518	0.705
Family history				
	Myocardial infarction	0.53947	1.819	0.767
	Stroke	1.77387	2.297	0.441
	Dyslipidemia	2.43881	1.782	0.172
	Diabetes	-1.07516	1.754	0.541
	Hypertension	-1.74105	1.774	0.327
	Obesity	-3.81356	1.833	0.039
Sociodemographic variables				
	Gender (Female)	-7.21638	1.687	0.00 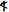
Age		-0.07133	0.113	0.529
Lifestyles				
	No physical activity peer week	-0.64469	1.750	0.714

WC: Waist circumference; BMI: Body mass index; hs-CRP: High sensitivity C reactive protein; HDL-C, High-density lipoprotein cholesterol; LDL-C, Low-density lipoprotein cholesterol; SBP: Systolic blood pressure; DBP: Diastolic blood pressure; HOMA-IR: Homeostatic Model Assessment of insulin resistance.

In the multivariate analysis (table 3), variables that emerged as significant predictors (p < 0.05) for serum zinc levels were total fat intake and log-plasma triglycerides in the whole group; total fat intake and age (a negative predictor with marginal significance) among men; and log-plasma triglyceride levels, log-hs-CRP levels (a negative predictor with marginal significance), and total fat intake among women.

**Table 3:** Multiple linear regression model^1^ with serum zinc levels as the outcome

Variable	**β coefficient**	Standard error	p value
% body fat	-0.07303	0.135	0.589
Log-Plasma plasma triglycerides	10.18376	3.901	0.010
Log-hs-CRP	-3.19418	3.162	0.314
Log-Glucose	1.72965	19.498	0.929
Total fat intake	-0.15065	0.031	**< 0.001**
Family history of obesity	-0.76590	1.805	0.672
Gender (Female)	-4.77600	2.683	0.076

hs-CRP: high sensitivity C reactive protein

^1^ The multiple linear regression model was built with variables related to serum zinc (p < 0.1) in a preliminary univariate analysis.

Table 4 shows the multiple models from table 3 adjusted for a preestablished set of covariates (age, calories per day, BMI, and physical activity). Findings were similar to those from the multiple models in table 3. The female gender improved its statistical significance as a negative predictor of serum zinc levels (p = 0.076 to p = 0.043).

**Table 4:** Serum zinc's explanatory multiple model^1^ adjusted for a preestablished set of covariates^2^

Variable	**β coefficient**	Standard error	p value
% body fat	-0.07303	0.135	0.589
Log-Plasma plasma triglycerides	10.18376	3.901	**0.010**
Log-hs-CRP	-3.19418	3.162	0.314
Log-Glucose	1.72965	19.498	0.929
Total fat intake	-0.15065	0.031	**< 0.001**
Family history of obesity	-0.76590	1.805	0.672
Gender (Female)	-4.77600	2.683	0.076

hs-CRP: high sensitivity C reactive protein

^1^ The multiple linear regression model was built with variables related to serum zinc (p < 0.1) in a preliminary univariate analysis.

## Discussion

The present study assessed the independent relationship of a group of nutritional, sociodemographic, biochemical, anthropometrical, and lifestyle variables with serum zinc levels. Female gender and variables related to cardiometabolic risk, such as plasma triglyceride levels and total fat intake, were associated with serum zinc levels.

We identified plasma triglycerides as the only lipid profile variable significantly and independently related to serum zinc levels in the entire group and among women. To our knowledge, this is the first report of a relationship between plasma triglycerides and serum zinc in a multivariate analysis. Previously, Tully *et al*. described a positive correlation of serum zinc with total and LDL cholesterol in elderly women (77-99 years old), and Laitinen *et al*. reported correlations with LDL-C, HDL-C, and total cholesterol in young people (3-18 years old) ^14^^,^^15^. However, He *et al*. did not find a relationship between serum zinc and lipid profile in men ^16^. Tully *et al*. supported their findings with red meat consumption, a source of zinc, and saturated fat. In contrast, our study found that the higher the total fat intake, the lower the serum zinc levels. We did not find similar studies evaluating total fat intake as a potential predictor of serum zinc levels.

Serum zinc levels could negatively respond to fat-load meals through the metal consumption in the peripheral tissues, as there is evidence of zinc as a promoter factor of insulin signaling ^17^. Another potential explanation for the inverse association between fat intake and zinc levels might lie in the pro- inflammatory effects of intracellular fat ^18^. Inflammation, in turn, triggers the sequestration of tissue zinc ^19^. Meanwhile, the positive association between plasma triglyceride levels and serum zinc could be related to the lipolytic effects of serum zinc-a2-glycoprotein ^20^^).^ However, these hypotheses and other possible mechanisms need further evaluation.

In this analysis, women presented lower zinc values than men, and female gender was a negative predictor of serum zinc levels. However, the relationship between serum zinc levels and gender is not conclusive. While our finding by gender aligns with those reported by Mariani *et al*. ^21^, other authors did not observe that relationship ^22^^,^^23^, and even Schumacher *et al*. had reported higher serum zinc levels in women ^24^. Although Andriollo- Sanchez *et al*. argued for the lack of difference in serum zinc by gender based on a low hormonal status in their middle-aged and old populations, the Mariani *et al*. study was conducted in healthy elderly subjects.

In our analysis, women presented a higher body fat percentage and hs- CRP levels than men, and these variables were inversely associated with serum zinc levels in univariate analysis. hs-CRP levels were significant serum zinc predictors in the multiple models if gender and body fat percentage were not included, while body fat percentage was found to be associated only if gender was not included in the multivariate analysis (data not shown). These findings are consistent with the hypothesis of a possible subclinical inflammation derived from adipose tissue as a modulator of serum zinc levels, as previously discussed.

Among the possible nutritional determinants of serum zinc levels, zinc intake was not a significant predictor. This finding is coherent with two explanations: First, serum zinc levels are not an ideal marker for assessing zinc status, except in cases of severe deficiency ^25^^,^^26^; and second, serum zinc levels are influenced or modulated by non-nutritional factors, as we found.

A non-significant correlation between zinc intake and serum zinc was described by Hyun *et al*. (r = 0.005; p = 0.929), but it became significant when zinc supplement consumption was added to zinc intake, although this correlation was weak (r = 0.114; p = 0.027) ^27^. Andriollo-Sanchez *et al*. also reported a weak but significant correlation between zinc intake and serum zinc (r = 0.129), but zinc intake was not discriminate between food and supplement consumption ^22^.

In agreement with our non-significant finding, Hennigar *et al*. did not find an association between zinc intake and serum levels of the same micronutrient in the U.S. general population ^3^. In contrast, some studies ^25^^,^^28^^,^^29^ have found significant increments in serum zinc levels after supplementation with zinc. It seems zinc supplement use could improve the relationship between intake and circulating levels, perhaps due to a better bioavailability of commercial zinc salts and a considerable increase in consumed zinc. However, bioavailability would depend on the type of supplement (*e.g.*, aqueous, tablets) and used salt (*e.g.*, zinc sulfate, histidinate) ^30^^,^^31^. The use of mineral or vitamin supplements was an exclusion criterion in our study, and therefore, the zinc intake is from nutritious sources.

There are limitations to the present study that need mentioning. The 24- hour recall survey covered only one day, and averaging data over two days might enhance the precision of individual intake estimations. Additionally, the study’s cross-sectional nature limits our ability to establish cause-effect relationships. The group of individuals in the original project constituted a convenience sample. Hence, our findings should be compared with results from future population-based studies among individuals without non-communicable chronic diseases. Similarly, excluding individuals with overweight/obesity would have been advisable due to the potential effects of excess adiposity on the relationship between cardiometabolic determinants and serum zinc.

However, in Colombia, one out of every two individuals is overweight or obese ^32^, and excluding individuals with this condition would significantly impact the sample size and the ability to detect associations. Nevertheless, when the obtained relationships were adjusted for BMI and other covariates, they did not substantially change.

On the other hand, the recruited sample in our study is a strength in terms of apparently healthy condition, and the absence of potential confounding factors (tobacco use, multivitamin supplements, and medication consumption). Similarly, the evaluation of a wide range of cardiovascular risk markers and family cardiometabolic disease history (not limited to nutritional and anthropometric variables) as predictors of serum zinc levels is another advantageous aspect of the present research.

In summary, variables related to cardiovascular risk, such as plasma triglyceride levels, total fat intake, and hs-CRP levels, were identified as predictors for serum zinc levels. Further studies are required to confirm our findings and to evaluate possible mechanisms for the relationship between serum zinc and predictor variables.

## References

[1] Baltaci AK, Yuce K, Mogulkoc R. (2018). Zinc metabolism and metallothioneins. Biol Trace Elem Res.

[2] Fernandez-Cao JC, Warthon-Medina M, Moran VH, Arija V, Doepking C, Serra-Majem L (2019). Zinc intake and status and risk of type 2 diabetes mellitus: A systematic review and metaanalysis. Nutrients.

[3] Hennigar SR, Lieberman HR, Fulgoni 3rd VL, McClung JP. (2018). Serum zinc concentrations in the US population are related to sex, age, and time of blood draw but not dietary or supplemental zinc. J Nutr.

[4] Chausmer AB. (1998). Zinc, insulin and diabetes. J Am Coll Nutr.

[5] Wiernsperger N, Rapin J. (2010). Trace elements in glucometabolic disorders: An update. Diabetol Metab Syndr.

[6] Little PJ, Bhattacharya R, Moreyra AE, Korichneva IL. (2010). Zinc and cardiovascular disease. Nutrition.

[7] Lohman TG. (1986). Applicability of body composition techniques and constants for children and youths. Exerc Sport Sci Rev.

[8] Lohman TG, Roche AF, Martorell R. (1988). Champaign, IL.

[9] Friedewald WT, Levy RI, Fredrickson DS. (1972). Estimation of the concentration of LDL in plasma, without use of the preparative ultracentrifuge. Clin Chem.

[10] Matthews DR, Hosker JP, Rudenski AS, Naylor BA, Treacher DF, Turner RC (1985). Homeostasis model assessment: Insulin resistance and beta-cell function from fasting plasma glucose and insulin concentrations in man. Diabetologia.

[11] Smith JCJ, Butrimovitz GP, Purdy WC. (1979). Direct measurement of zinc in plasma by atomic absorption spectroscopy. Clin Chem.

[12] Quintero S, Escobar E. (2001). Tabla de composición de alimentos.

[13] Lang TA, Secic M. (2006). How to report statistics in medicine: Annotated guidelines for authors, editors, and reviewers.

[14] Laitinen R, Vuori E, Viikari J. (1989). Serum zinc and copper: Associations with cholesterol and triglyceride levels in children and adolescents. Cardiovascular risk in young Finns. J Am Coll Nutr.

[15] Tully CL, Snowdon DA, Belcher JD. (1996). Serum zinc and plasma lipoproteins in elderly women: Findings from the nun study. J Trace Elem Exp Med.

[16] He JA, Tell GS, Tang YC, Mo PS, He GQ. (1992). Relation of serum zinc and copper to lipids and lipoproteins: The Yi People Study. J Am Coll Nutr.

[17] Norouzi S, Adulcikas J, Sohal SS, Myers S. (2018). Zinc stimulates glucose oxidation and glycemic control by modulating the insulin signaling pathway in human and mouse skeletal muscle cell lines. PLoS One.

[18] De Souza CT, Araujo EP, Bordin S, Ashimine R, Zollner RL, Boschero AC (2005). Consumption of a fat-rich diet activates a proinflammatory response and induces insulin resistance in the hypothalamus. Endocrinology.

[19] Tapiero H, Tew KD. (2003). Trace elements in human physiology and pathology: Zinc and metallothioneins. Biomed Pharmacother.

[20] Xiao X, Li H, Qi X, Wang Y, Xu C, Liu G (2017). Zinc alpha 2 glycoprotein alleviates palmitic acid-induced intracellular lipid accumulation in hepatocytes. Mol Cell Endocrinol.

[21] Mariani E, Cornacchiola V, Polidori MC, Mangialasche F, Malavolta M, Cecchetti R (2006). Antioxidant enzyme activities in healthy old subjects: Influence of age, gender and zinc status. Biogerontology.

[22] Andriollo-Sanchez M, Hininger-Favier I, Meunier N, Toti E, Zaccaria M, Brandolini-Bunlon M (2005). Zinc intake and status in middle-aged and older European subjects: The ZENITH study. EurJClin Nutr.

[23] Romero CD, Sánchez PH, Blanco FL, Rodríguez Rodríguez E, Serra Majem L. (2002). Serum copper and zinc concentrations in a representative sample of the Canarian population. J Trace Elem Med Biol.

[24] Schumacher M, JL D, Corbella J. (1994). Zinc and copper levels in serum and urine: Relationship to biological, habitual and environmental factors. Sci Total Environ.

[25] Brown KH, Peerson JM, Rivera J, Allen LH. (2002). Effect of supplemental zinc on the growth and serum zinc concentrations of prepubertal children: A meta-analysis of randomized controlled trials. Am J Clin Nutr.

[26] Wood RJ. (2000). Assessment of marginal zinc status in humans. J Nutr.

[27] Hyun TH, Barrett-Connor E, Milne DB. (2004). Zinc intakes and plasma concentrations in men with osteoporosis: The Rancho Bernardo Study. Am J Clin Nutr.

[28] Bogden JD, Oleske JM, Lavenhar MA, Munves EM, Kemp FW, Bruening KS (1988). Zinc and immunocompetence in elderly people: Effects of zinc supplementation for 3 months. Am J Clin Nutr.

[29] Gunasekara P, Hettiarachchi M, Liyanage C, Lekamwasam S. (2011). Effects of zinc and multimineral vitamin supplementation on glycemic and lipid control in adult diabetes. Diabetes Metab Syndr Obes.

[30] Schölmerich J, Freudemann A, Köttgen E, Wietholtz H, Steiert B, Löhle E (1987). Bioavailability of zinc from zinc-histidine complexes. I. Comparison with zinc sulfate in healthy men. Am J Clin Nutr.

[31] Solomons NW, Romero-Abal ME, Weiss G, Michalke B, Schumann K. (2011). Bioavailability of zinc from NutriSet zinc tablets compared with aqueous zinc sulfate. ur J Clin Nutr.

[32] Jimenez-Mora MA, Nieves-Barreto LD, Montano-Rodriguez A, Betancourt-Villamizar EC, Mendivil CO. (2020). Association of overweight, obesity and abdominal obesity with socioeconomic status and educational level in Colombia. Diabetes Metab Syndr Obes Targets Ther.

